# Interleukins 6 and 15 Levels Are Higher in Subcutaneous Adipose Tissue, but Obesity Is Associated with Their Increased Content in Visceral Fat Depots

**DOI:** 10.3390/ijms161025817

**Published:** 2015-10-28

**Authors:** Marta Izabela Jonas, Alina Kurylowicz, Zbigniew Bartoszewicz, Wojciech Lisik, Maurycy Jonas, Zbigniew Wierzbicki, Andrzej Chmura, Piotr Pruszczyk, Monika Puzianowska-Kuznicka

**Affiliations:** 1 Department of Human Epigenetics, Mossakowski Medical Research Centre, Polish Academy of Sciences, 5 Pawinskiego Street, 02-106 Warsaw, Poland; E-Mails: martajonas@poczta.onet.pl (M.I.J.); zbigniew.bartoszewicz@wum.edu.pl (Z.B.); 2 Department of General and Transplantation Surgery, Medical University of Warsaw, 59 Nowogrodzka Street, 02-005 Warsaw, Poland; E-Mails: wojciech.lisik@wum.edu.pl (W.L.); morjon@poczta.onet.pl (M.J.); zbigniew-wierzbicki@wp.pl (Z.W.); andrzejchmura@wum.edu.pl (A.C.); 3 Department of Internal Medicine and Cardiology, Medical University of Warsaw, Lindleya 4, 02-005 Warsaw, Poland; E-Mail: piotrpruszczyk@wum.edu.pl; 4 Department of Geriatrics and Gerontology, Medical Centre of Postgraduate Education, 99 Marymoncka Street, 01-813 Warsaw, Poland

**Keywords:** obesity, inflammation, interleukins, subcutaneous adipose tissue, visceral adipose tissue

## Abstract

Excess adiposity is associated with chronic inflammation, which takes part in the development of obesity-related complications. The aim of this study was to establish whether subcutaneous (SAT) or visceral (VAT) adipose tissue plays a major role in synthesis of pro-inflammatory cytokines. Concentrations of interleukins (IL): 1β, 6, 8 and 15 were measured at the protein level by an ELISA-based method and on the mRNA level by real*-*time PCR in VAT and SAT samples obtained from 49 obese (BMI > 40 kg/m^2^) and 16 normal-weight (BMI 20–24.9 kg/m^2^) controls. IL-6 and IL-15 protein concentrations were higher in SAT than in VAT for both obese (*p* = 0.003 and *p* < 0.0001, respectively) and control individuals (*p* = 0.004 and *p* = 0.001, respectively), while for IL-1β this was observed only in obese subjects (*p* = 0.047). What characterized obese individuals was the higher expression of IL-6 and IL-15 at the protein level in VAT compared to normal-weight controls (*p* = 0.047 and *p* = 0.016, respectively). Additionally, obese individuals with metabolic syndrome had higher IL-1β levels in VAT than did obese individuals without this syndrome (*p* = 0.003). In conclusion, concentrations of some pro-inflammatory cytokines were higher in SAT than in VAT, but it was the increased pro-inflammatory activity of VAT that was associated with obesity and metabolic syndrome.

## 1. Introduction

Obesity is a disease leading to serious complications that reduce the quality of life and might shorten its duration. The molecular mechanisms of obesity-related complications are not fully understood; it is suggested that inflammation may play an important role. Indeed, a low-grade chronic inflammatory state, also called metaflammation, is present in obese individuals and negatively impacts the function of multiple organs and tissues [1]. Elevated levels of inflammatory markers correlate with the risk of insulin resistance, cardiovascular disease, diabetes, and dyslipidemia, and stimulate infiltration by lymphocytes into the endothelium as well as vascular smooth muscle cells migration, promoting intimal thickening and atherosclerosis [2,3,4,5]. In accordance with these observations, data obtained on animal models show that the administration of anti-inflammatory agents may prevent the development of metabolic syndrome [6].

One area where many unknowns still remain is the origin of excess inflammatory cytokines in obese individuals. *In vitro* experiments suggest that initiation of the inflammatory process in response to an excess of nutrients takes place in the adipose tissue itself. According to this theory, the accumulation of lipids leads to increased expression of genes encoding cytokines, chemokines, and adhesion molecules in adipocytes, attracting infiltrating immune cells that contribute to the synthesis of pro-inflammatory mediators [1,7,8,9]. Since it was found that subcutaneous (SAT) and visceral (VAT) adipose tissue depots differ in terms of their metabolic activity, a number of studies were conducted to assess which of them plays a dominant role in the development of chronic inflammation [10,11,12,13]. Morphological studies comparing the intensity of inflammatory infiltration in different adipose tissue depots showed that in both obese and in normal-weight individuals, VAT samples contain more macrophages than SAT [9,14,15]. However, results of studies regarding the concentration of cytokines in adipose tissues in different locations are unequivocal and their conclusions are predominantly based on the analysis of mRNA levels [16,17,18]. Therefore, to establish whether subcutaneous or visceral adipose tissue plays a major role in the development of obesity-associated inflammation, we analyzed the concentration of pro-inflammatory cytokines directly in the VAT and SAT samples obtained from obese individuals and from normal-weight controls. Out of many candidate genes implicated in the inflammatory milieu in adipose tissue, we selected four interleukins (IL): IL-1β, IL-6, IL-8 and IL-15. Our choice was dictated by their documented *in vitro* and *in vivo* involvement in the pathogenesis of obesity-related complications [5,19,20,21,22,23], as well as previous reports of their elevated concentrations in sera [10,24,25] and/or mRNA levels in the adipose tissue of obese individuals [16,17,24,26]. Of note is that until now there were only few reports regarding direct measurement of these cytokines in adipose tissue at the protein level [24,27].

## 2. Results

### 2.1. Expression of Cytokines in Adipose Tissues from Obese and Normal-Weight Individuals

The initial analysis showed that the mean levels of the analyzed cytokines did not differ in the adipose tissue of males and females; therefore, all subsequent analyses were performed for all study participants together.

**Figure 1:**
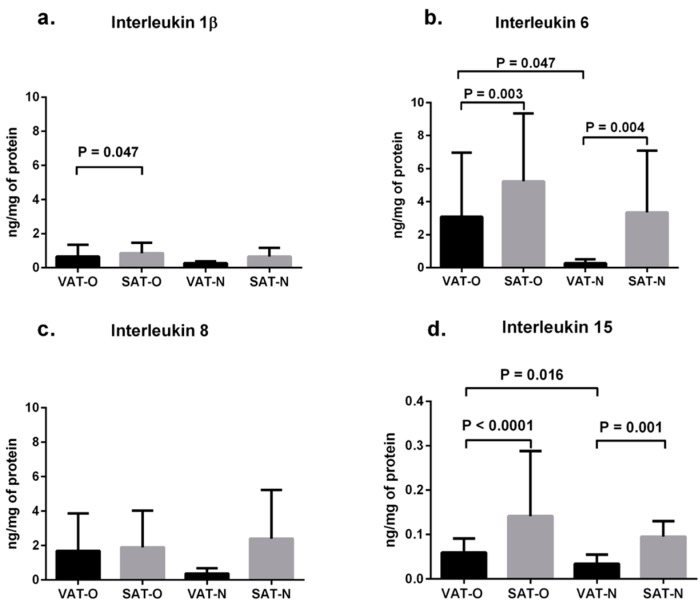
Mean protein levels of interleukin 1β (**a**); interleukin 6 (**b**); interleukin 8 (**c**) and interleukin 15 (**d**) in the visceral (VAT) and subcutaneous (SAT) adipose tissues of obese (O) and normal-weight (N) individuals. Results are shown as mean ± standard deviation.

The mean IL-6 protein concentrations (Figure 1b) were higher in SAT than in VAT both in obese individuals (5.23 *vs.* 3.09 ng per 1 mg of total protein, *p* = 0.003) and in normal-weight subjects (3.35 *vs.* 0.27 ng per 1 mg of total protein, *p* = 0.004). Similarly, the mean IL-15 levels (Figure 1d) were higher in SAT than in VAT for both weight groups (0.14 *vs.* 0.06 ng per 1 mg of total protein, *p* < 0.0001, and 0.09 *vs.* 0.03 ng per 1 mg of total protein, *p* = 0.001, respectively). The mean concentration of IL-1β was also higher in SAT than in VAT of obese study subjects (0.86 *vs.* 0.67 ng per 1 mg of total protein, *p* = 0.047), while in normal-weight subjects the difference between SAT and VAT was not significant (Figure 1a). No differences were observed in IL-8 concentrations between the investigated tissues (Figure 1c).

The mean protein concentrations of the investigated cytokines were higher in adipose tissues obtained from obese than from normal-weight individuals, but the differences were significant only for VAT content of IL-6 (3.09 *vs.* 0.27 ng per 1 mg of total protein, *p* = 0.047, Figure 1b) and IL-15 (0.06 *vs.* 0.03 ng per 1 mg of total protein, *p* = 0.016, Figure 1d).

### 2.2. Expression of Cytokine mRNA Levels in Adipose Tissues from Obese and Normal-Weight Individuals

Initial analysis showed that the mean IL-1β, IL-6, IL-8, and IL-15 mRNA levels did not differ in the adipose tissue of males and females, and all further analyses were performed for all study subjects together.

**Figure 2:**
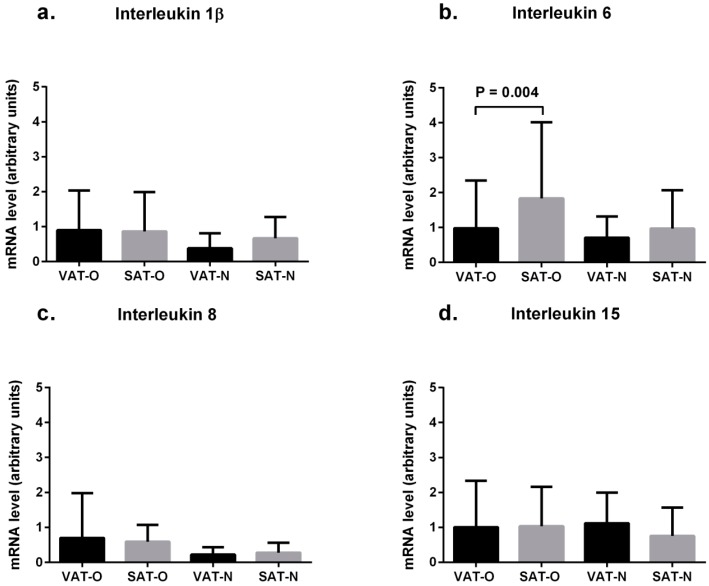
Mean interleukin 1β (**a**), interleukin 6 (**b**) interleukin 8 (**c**) and interleukin 15 (**d**) mRNA levels in the visceral (VAT) and subcutaneous (SAT) adipose tissues of obese (O) and normal-weight (N) individuals. Results, normalized against the expression of *ACTB*, are shown as mean ± standard deviation.

The mean IL-6 mRNA levels were higher in SAT than in VAT of obese individuals (1.83 *vs.* 0.98 arbitrary units, *p* = 0.004, Figure 2b). This was not observed in normal-weight subjects. No significant differences in the mean mRNA levels of IL-1β, IL-8, and IL-15 between the adipose tissue types were observed (Figure 2).

Notably, the mean mRNA levels of the investigated cytokines did not differ between the tissues obtained from obese and from normal-weight patients (Figure 2).

**Figure 3:**
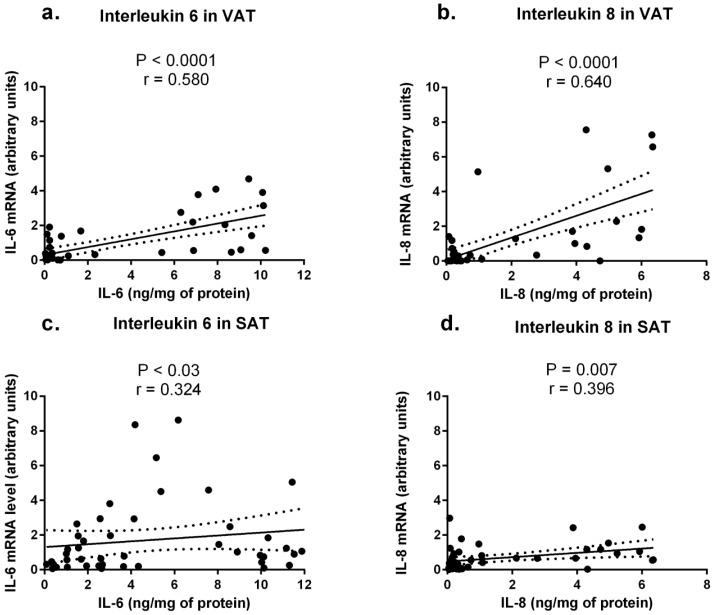
Correlation of interleukin 6 and interleukin 8 protein and mRNA levels in the visceral (VAT) and subcutaneous (SAT) adipose tissues.

After pooling the results from obese and normal-weight individuals, a significant positive correlation between mRNA and protein concentrations of IL-6 and of IL-8 were found in VAT (*r* = 0.580, *p* < 0.0001, for IL-6 and *r* = 0.640, *p* < 0.0001 for IL-8, Figure 3a,b) and in SAT (*r* = 0.324, *p* = 0.03 for IL-6 and *r* = 0.396, *p* = 0.007 for IL-8, Figure 3c,d). No mRNA-protein correlations for IL-1β and IL-15 were found.

### 2.3. Cytokine Concentrations in Sera from Obese and Normal-Weight Individuals and Their Correlation with Protein Levels in Adipose Tissues

The mean serum IL-6 protein concentration was higher in obese patients than in normal-weight individuals (1.59 pg/mL *vs.* 0.68 pg/mL) with a borderline *p* value of 0.08. No significant differences were found between the obese and normal-weight subjects in the mean serum concentrations of IL-1β, IL-8, and IL-15 proteins.

A significant positive correlation between the IL-6 serum levels and its protein concentration in the SAT of obese individuals was observed (*p* = 0.042, *r* = 0.292). No other correlations were detected.

### 2.4. Correlation between Cytokine Concentrations and Clinical and Biochemical Parameters in Obese Individuals

Serum as well as VAT and SAT levels of IL-1β, IL-6, IL-8, and IL-15 were correlated with basic biochemical and clinical parameters and with the presence of obesity-related complications (listed in Table 1), but no significant correlations were observed. When the obese study participants were stratified according to the presence or absence of metabolic syndrome (MS), we found that individuals diagnosed with MS had significantly higher VAT levels of IL-1β (0.88 *vs.* 0.29 ng per 1 mg of total protein, *p* = 0.003). Mean levels of IL-6 and IL-15 were also higher with a borderline significance in VAT of MS patients (4.07 *vs.* 1.43 ng per 1 mg of total protein, *p* = 0.058 for IL-6 and 0.063 *vs.* 0.043 ng per 1 mg of total protein, *p* = 0.07 for IL-15).

## 3. Discussion

Obesity is accompanied by chronic low-grade inflammation, the source of which seems to be the adipose tissue itself; it is unclear, however, which adipose tissue depot, subcutaneous or visceral, plays a dominant role in the synthesis of pro-inflammatory mediators. Correlation of cytokine serum levels and/or the prevalence of obesity-associated co-morbidities with the distribution of adipose tissue [16,28,29] or with mRNA levels of various cytokines measured in VAT or SAT [16,17,18,30] did not resolve this issue. Therefore, in this work we measured concentrations of four pro-inflammatory interleukins: IL-1β, IL-6, IL-8, and IL-15, directly in adipose tissues. We correlated the results with the relevant clinical and biochemical parameters as well as with the presence of inflammation-related co-morbidities.

Our results point to abdominal SAT as a more potent locus of cytokine synthesis than VAT, since we found that concentrations of IL-6 and IL-15 were significantly higher in SAT than in VAT of both obese and normal-weight individuals, and the concentration of IL-1β was also higher in the SAT of obese subjects. On the other hand, in our study, obesity was associated with increased pro-inflammatory activity of VAT since the VAT of obese subjects contained significantly more IL-6 and IL-15 than the VAT of normal-weight individuals, while the difference in cytokine content in SAT of both weight groups was not significant. This finding is in concordance with the observations linking central adiposity with higher inflammatory rates [10,11,12] and with a higher risk of metabolic complications including insulin resistance, hyperlipidemia, and hypertension [13,16,30,31]. Moreover, in cell cultures of adipocytes originating from obese individuals, cells derived from VAT released more pro-inflammatory mediators (including IL-1β and IL-6) than subcutaneous adipocytes [32,33,34,35,36]. In turn, in animal models of obesity, surgical removal of visceral fat led to an increase in insulin sensitivity and amelioration of diet-induced diabetes [37]. Our finding that obese individuals who have met the diagnostic criteria for metabolic syndrome had higher mean levels of IL-1β, IL-6, and IL-15 in VAT than obese individuals without MS also supports this hypothesis.

There are reports, however, that question the dominant role of VAT in the development of metaflammation, including a recent study showing that the mRNA levels of several pro-inflammatory interleukins were higher in SAT than in VAT of obese patients [17]. Indeed, central adiposity is commonly associated with high amounts of abdominal SAT, which has a unique gene expression profile, different from SAT collected from other depots [13,38,39]. Differences in gene expression result in higher metabolic activity of abdominal SAT compared to gluteofemoral depots which is manifested, for example, by increased postprandial fatty acid uptake and higher lipolytic activity in response to hormonal stimuli [40,41]. Our findings that abdominal SAT is a predominant site of at least some of pro-inflammatory cytokine synthesis, and that the correlation between IL-6 serum and SAT concentrations is observed only in the obese, support the view that increased amount of SAT in the obese might also contribute to metaflammation observed in obesity.

Moderately increased IL-6 levels seen in obese individuals may contribute to the development of obesity-associated complications, e.g., insulin resistance, via the inhibition of lipoprotein lipase activity in the adipose tissue [21,42]. It is estimated that 15%–35% of total IL-6 in blood originates from adipose tissue [43], and our results point to SAT as a more potent source of this cytokine in both obese and normal-weight individuals. On the other hand, in our study, obesity was associated with increased synthesis of IL-6 in VAT, which is in agreement with the results of studies assessing IL-6 concentrations at the mRNA level, including micro-array studies [44,45], as well as the results of *in vitro* experiments showing that adipocytes isolated from VAT of obese individuals release more IL-6 than those from normal-weight subjects [34,35]. In the only other study where IL-6 concentration was assessed directly in SAT of obese subjects by ELISA method, weight loss was associated with a decrease of its levels [24].

IL-15 expression profile in adipose tissues resembled that of IL-6. IL-15 is a classic pro-inflammatory cytokine involved in both innate and adaptive immune responses, produced by multiple types of cells, including adipocytes [46]. Results of studies on the relationship between IL-15 serum concentration and body composition in humans are ambiguous: some authors observed a negative correlation between IL-15 serum levels with trunk and total fat mass [22], while others (including this study) recorded no differences in circulating IL-15 between lean and obese individuals [27]. Sources of circulating IL-15 in obese humans remain unknown. Until now, a systematic study of IL-15 mRNA and protein expression in various depots of fat tissue has not been performed [23]. The only study assessing the concentration of this cytokine directly in 20 samples of human SAT using the microdialysis method revealed that its levels in obese individuals were higher compared to lean subjects [27], which is consistent with the results of our study obtained using ELISA method, although the difference observed by us did not reach the level of significance. Notably, in our study we also examined VAT and observed a higher level of this cytokine in obese subjects than in those of normal weight. Moreover, we show here that the increased IL-15 levels in VAT of obese individuals correlates with presence of metabolic syndrome.

*In vitro* studies as well as animal models proved that excess accumulation of saturated free fatty acids in adipose tissues of obese individuals triggers NLRP3 (NOD- and pyrin domain-containing like receptor 3) inflammasome activation which mediates the formation of mature forms of IL-1β [47]. This cytokine, acting via IL-1 receptor, accelerates generation of other pro-inflammatory mediators initiating a self-amplifying cytokine network and plays a key role in the pathogenesis of pancreatic islet inflammation [48]. Indeed, serum concentrations of IL-1β were found to be elevated in obese individuals, and correlated with insulitis and diabetes [19], while neutralization of IL-1β action via IL-1 receptor antagonist improves metabolic parameters in type 2 diabetes [49]. However, results of studies where IL-1β expression was measured at the mRNA level in adipose tissues of obese individuals were inconclusive: in some studies, IL-1β mRNA levels were found to be higher in VAT than in SAT and diminished significantly after bariatric surgery-induced weight loss [26], while other authors observed an opposite trend [17]. In obese individuals investigated in our study, IL-1β protein levels in VAT were higher compared to normal-weight patients, especially in those diagnosed with metabolic syndrome, further supporting the role of this cytokine in the development of obesity-associated complications [19]. Importantly, we did not observe a correlation between concentrations of IL-1β measured at the mRNA and protein levels in the investigated tissues.

IL-1β acting in a paracrine manner in adipocytes stimulated the production of IL-8, a potent chemoattractant involved in the adhesion of monocytes to endothelium and in the migration of vascular smooth cells, proposed therefore as a mediator between obesity and atherosclerosis [50]. The mean values of plasma IL-8 were found to be increased in obese individuals and correlated with VAT volume [25]. In turn, Spoto *et al.* showed that IL-8 mRNA levels were significantly higher in SAT than in VAT and correlated negatively with its plasma concentrations [17]. In our study, such a correlation was not confirmed at the protein level. We also found no difference in IL-8 protein levels in different fat depots. This finding suggests that a higher level of IL-8 observed in some populations of obese patients might not originate from the adipose tissue.

In summary, we report here that even though concentrations of the important pro-inflammatory cytokines are higher in subcutaneous than in visceral fat depots, obesity and metabolic syndrome are associated with an increased pro-inflammatory activity of VAT. Notably, cytokine concentrations assessed directly in adipose tissues did not always correspond to their mRNA levels in the same tissues, emphasizing the need of verifying mRNA expression results at the protein level.

## 4. Material and Methods

### 4.1. Studied Groups

The group of obese individuals with a body mass index (BMI, calculated as weight in kg divided by height squared, m^2^) >40 kg/m^2^ consisted of 49 patients (44 females and 5 males) whose basic clinical characteristics, biochemical parameters, and co-morbidities are summarized in Table 1. In this group the metabolic syndrome (MS) was diagnosed on the basis of the International Diabetes Federation criteria for the Europeans [51] in 28 (57.1%) individuals.

**Table 1:** Selected clinical and biochemical parameters of study participants.

Parameter	Obese Individuals		Normal-Weight Controls		*p*
	Mean ± SD	Min–Max	Mean ± SD	Min–Max	
Age (years)	42.02 ± 10.33	20–59	44.08 ± 13.50	26–63	0.623
Weight (kg)	132.17 ± 18.61	100–198.6	67.92 ± 10.48	54–90	<0.0001
BMI (kg/m^2^)	47.02 ± 4.91	40.48–59.26	23.06 ± 1.77	20.07-24.93	<0.0001
Adipose tissue (% body mass)	48.55 ± 4.10	36.30–57.23	–	–	
Waist circumference (cm)	124.47 ± 18.78	97–167	–	–	
CRP (nmol/L)	123.15 ± 114	11.43–657.16	30.86 ± 16.38	6.67–49.52	0.04
Glucose (mmol/L)	5.65 ± 1.53	3.36–10.08	5.17 ± 0.21	4.77–5.44	0.314
Total cholesterol (mmol/L)	5.20 ± 1.03	3.13–7.86	4.80 ± 0.20	4.62–4.92	0.623
LDL (mmol/L)	3.28 ± 1.06	1.25–5.64	2.62 ± 0.08	2.75–2.80	0.547
HDL (mmol/L)	1.24 ± 0.23	0.78–1.78	1.47 ± 0.27	1.24–1.76	0.136
Triglycerides (mmol/L)	1.44 ± 0.73	0.52–2.92	1.33 ± 0.09	1.10–1.46	0.797
Co-morbidities					
Type 2 DM/IGT	24 (49.0%)		none		
Hypertension	29 (59.2%)		none		
Hyperlipidemia	30 (61.2%)		none		

BMI: body mass index calculated as weight (kg) divided by height squared (m^2^); LDL: low density lipoproteins; HDL: high density lipoproteins; CRP: C-reactive protein; DM: diabetes mellitus; IGT: impaired glucose tolerance.

The control group of normal-weight patients (BMI 20–24.9 kg/m^2^) consisted of 16 individuals (11 females and 5 males) undergoing elective surgical procedures. Apart from cholelithiasis (without signs of inflammation) or inguinal hernia, they had no history of any chronic disease, including components of MS. Their normal health status was confirmed by physical examination and blood tests (Table 1). Although their adipose tissue content was not calculated, based on their medical history, BMI, biochemical parameters, as well as absence of any components of metabolic syndrome, they were considered to be metabolically healthy. None of the participants received anti-inflammatory treatment.

Before surgery, all participants underwent a medical examination and gave 15 mL of venous blood. Sera obtained from these samples were stored at −80 °C until the biochemical and immunological measurements were performed. Adipose tissue samples were immediately frozen at −80 °C and homogenized in liquid nitrogen. The study was approved by the Bioethics Committee of the Medical University of Warsaw and written informed consent was obtained from all participants.

### 4.2. Adipose Tissues

Pairs of visceral (*n* = 49) and subcutaneous (*n* = 49) adipose tissues were obtained from obese patients during bariatric surgery. Control tissues were collected from 23 normal-weight patients undergoing elective cholecystectomy (14 samples of VAT and 4 samples of SAT) or operated on for inguinal hernia (five samples of SAT). All samples of SAT were collected from the abdominal region.

### 4.3. Isolation of a Protein Fraction from Adipose Tissue

To liquefy the tissues, a buffer containing 10 mM phosphate-buffered saline (pH = 7.4), 138 mM sodium chloride, 2.7 mM potassium chloride, 2.5 mM ethylenediaminetetraacetic acid, 1 mM sodium orthovanadate, 1% Nonindet, 1% Triton X-100 (all reagents from Sigma-Aldrich, Saint Louis, MO, USA), Pierce phosphatase inhibitor (Thermo Fisher Scientific, Rockford, IL, USA), and a cocktail of protease inhibitors (cOmplete, Roche Diagnostic, Mannheim, Germany) were used. Buffer (1.5 mL, cooled down to 4 °C) was added to 300 mg of the adipose tissue previously homogenized in liquid nitrogen and the mixture was again homogenized for 3 min at 4 °C, then centrifuged for 30 min at 4 °C, 10,000× *g*. The middle layer of the supernatant was collected and centrifuged again under the same conditions. The supernatant obtained after the second centrifugation was used for measuring the total protein concentration with the Pierce BCA Protein Assay Kit (Thermo Fisher Scientific, Rockford, IL, USA) and for measuring cytokine concentrations.

### 4.4. Measurement of Cytokine Concentrations

To measure IL-1β, IL-6, IL-8, and IL-15 concentrations in serum and in protein extracts from adipose tissues, an ELISA-based chemiluminescent custom-made Q-plex Custom array (Quansys Bioscience, West Logan, UT, USA) was used. Luminescence was assessed in the Molecular Imager Versa Doc™ MP 5000 System (Bio-Rad, Hercules, CA, USA) according to the manufacturer’s guidelines. Results were analyzed with the Q-View software version 2.17 (Quansys Bioscience, West Logan, UT, USA). Measurements of interleukin concentrations in adipose tissues were normalized to total protein concentration in protein extracts obtained from these tissues. The mean protein concentrations in extracts from visceral (VAT) and subcutaneous (SAT) tissues obtained from obese (O) and normal-weight (N) individuals were similar (*p* > 0.05, Supplementary Figure S1).

### 4.5. Isolation of Total RNA, Reverse Transcription, and Real-Time PCR

Approximately 500 mg of each tissue (homogenized in liquid nitrogen) was used for the total RNA extraction with TRIzol Reagent (Invitrogen, Carlsbad, CA, USA) according to the protocol provided by the manufacturer. Next, 100 ng of each RNA was used for reverse transcription, performed with RevertAid First Strand cDNA Synthesis Kit (Fermentas, Vilnius, Lituania). The obtained cDNA was diluted in RNAse-free dH_2_O and subsequently used as a template in real-time PCR performed in LightCycler 480 Instrument II (Roche, Mannheim, Germany) with specific primers (Table 2) and with LightCycler 480 Sybr Green I Master Kit (Roche, Mannheim, Germany). The PCR conditions were as follows: 1 cycle at 95 °C for 10 min, 40 cycles of 95 °C for 12 s, 55–61 °C for 12 s, 72 °C for 12 s followed by one melting curve cycle. All measurements were performed in triplicate. The results were normalized against the expression of the β-actin gene and presented in arbitrary units (AU) as mean mRNA levels.

**Table 2:** Primers used for the analysis of cytokine expression on mRNA level in adipose tissues.

Gene	Gene Description	Gene Bank	Primers		Annealing (°C)
*IL1B*	interleukin 1β	NM_000576	F	5ʹ-CACCAAGCTTTTTTGCTGTGAGT-3ʹ	60
			R	5ʹ-GCACGATGCACCTGTACGAT-3ʹ	
*IL6*	interleukin 6	NM_000600	F	5ʹ-CCTTCGGTCCAGTTGCCTTC-3ʹ	60
			R	5ʹ-GTGGGGCGGCTACATCTTTG-3ʹ	
*IL8*	interleukin 8	NM_000584	F	5ʹ-CACCGGAAGAACCATCTCACT-3ʹ	60
			R	5ʹ-TCAGCCCTCTTCAAAAACTTCTCC-3ʹ	
*IL15*	interleukin 15	NM_172175	F	5ʹ-GGATTTACCGTGGCTTTGAGTAATGAG-3ʹ	55
			R	5ʹ-GAATCAATTGCAATCAAGAAGTG-3ʹ	
*ACTB*	β-actin	NM_001101	F	5ʹ-CAGCCTGGATAGCAACGTAC-3ʹ	61
			R	5ʹ-TTCTACAATGAGCTGCGTGTG-3ʹ	

F: forward primer; R: reverse primer.

### 4.6. Statistical Analysis

The Student’s *t*/Mann–Whitney *U* test or Kruskal–Wallis analysis of variance were used to compare the differences in cytokine concentrations on protein and mRNA levels, while the Spearman correlation test was used to perform correlations between quantitative values. Normality of distribution was checked with the Shapiro-Wilk test, whilst homogeneity of the variance with the Levene’s test. The statistical analysis was performed with the Statistica software package v.10 (StatSoft, Tulsa, OK, USA).

## Supplementary Materials

Supplementary materials can be found at http://www.mdpi.com/1422-0067/16/10/25817/s1.
