# Supplementary material for: Interleukins 6 and 15 Levels Are Higher in Subcutaneous Adipose Tissue, but Obesity Is Associated with Their Increased Content in Visceral Fat Depots
Source: Int J Mol Sci. 2015 Oct 28;16(10):25817–30. doi: 10.3390/ijms161025817 (PMC4632828; doi:10.3390/ijms161025817)
Supplement: Supplementary file 1 [file ijms-16-25817-s001.pdf]

Supplementary Information

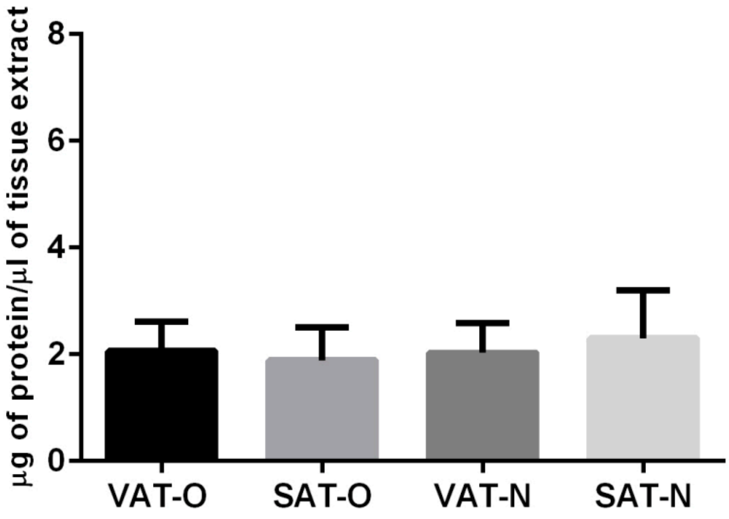

Figure S1. Mean total protein concentrations in visceral (VAT) and subcutaneous (SAT) adipose tissues extracts from obese (O) and normal-weight (N) individuals.
